# Longitudinal renal function following [¹⁷⁷Lu]Lu-PSMA I&T in metastatic castration-resistant prostate cancer

**DOI:** 10.1007/s00259-026-07893-4

**Published:** 2026-04-21

**Authors:** Matthew Cockcroft, Tom Ferguson, Katie Meehan, Zeyad Al-Ogaili

**Affiliations:** 1 Medical Oncology Department, Fiona Stanley Hospital, Perth, WA Australia; 2 Translational Oncology, Harry Perkins Institute for Medical Research, Perth, WA Australia; 3 Centre for Medical Research, University of Western Australia, Perth, WA Australia; 4 Nuclear Medicine Department, Fiona Stanley Hospital, Perth, WA Australia

**Keywords:** Prostate-specific membrane antigen (PSMA), Radioligand therapy, Lutetium-177, Renal function, Estimated glomerular iltration rate (eGFR), Nephrotoxicity, Metastatic castration-resistant prostate cancer (mCRPC)

## Abstract

**Purpose:**

The kidneys are recognised organs at risk during prostate-specific membrane antigen (PSMA)–targeted radioligand therapy. While clinically overt nephrotoxicity appears uncommon in late-line metastatic castration-resistant prostate cancer (mCRPC), the longitudinal trajectory of renal function following [¹⁷⁷Lu]Lu-PSMA I&T remains incompletely characterised. We aimed to quantify longitudinal changes in renal function in a treated mCRPC cohort and explore clinical factors associated with renal decline.

**Methods:**

This retrospective cohort study included patients with mCRPC treated with [¹⁷⁷Lu]Lu-PSMA I&T between November 2018 and July 2024. Longitudinal renal analyses were performed in patients with baseline and post-treatment estimated glomerular filtration rate (eGFR) measurements, calculated using the 2021 CKD-EPI creatinine equation (race-free version). Changes in eGFR over time were modelled using linear mixed-effects models with random intercepts and slopes. Fixed effects included time since first treatment, estimated cumulative renal absorbed dose (derived from a population-based conversion factor in the absence of patient-specific dosimetry), and a predefined Renal Risk Index (RRI) capturing baseline chronic kidney disease, hypertension, and diabetes mellitus. Additional analyses examined reconstructed pre-treatment trajectories using piecewise modelling, restricted 12-month analyses, and non-linearity testing. Clinically significant renal decline was defined as a ≥ 25% reduction in eGFR from baseline and evaluated using time-to-event analyses.

**Results:**

A total of 105 patients were included, contributing 238 post-treatment eGFR measurements over follow-up of up to 48 months. Renal function declined over time with an estimated mean decrease of 0.85 mL/min/1.73 m² per month (95% CI − 1.30 to − 0.40; *p* < 0.001). Baseline renal vulnerability was independently associated with lower absolute eGFR values (− 6.76 mL/min/1.73 m² per RRI unit; *p* < 0.001) but did not modify the rate of decline. Within the administered activity range and using population-based dose estimates, no clear dose–response relationship was detected; this finding should be interpreted in the context of the narrow activity range studied and the absence of patient-specific dosimetry. Reconstructed pre-treatment trajectories demonstrated stable renal function prior to therapy (β = +0.06 mL/min/month; *p* = 0.30), whereas post-treatment eGFR declined significantly (β = −0.60 mL/min/month; *p* < 0.001). Restricted analyses limited to the first 12 months showed similar slopes, with no evidence of non-linearity. Clinically significant renal decline occurred in 16 patients (15%), most frequently within the first year following treatment initiation.

**Conclusion:**

In this retrospective mCRPC cohort treated with [¹⁷⁷Lu]Lu-PSMA I&T, a measurable decline in renal function was observed over time, although the observational design precludes causal attribution to radioligand therapy. Clinically significant renal deterioration remained relatively uncommon. Baseline renal vulnerability influenced overall renal reserve but not the rate of decline, and no clear dose–response relationship was detected within the administered activity range. Extrapolation of these findings to earlier-line settings should be considered hypothesis-generating. These findings support baseline renal assessment and longitudinal monitoring as PSMA-targeted radioligand therapy expands to earlier disease settings.

## Background

The kidneys are recognised as key organs at risk during radioligand therapy because of their efficient glomerular filtration and subsequent tubular handling of small radiolabelled molecules. PSMA-targeted radioligands are freely filtered at the glomerulus and may undergo proximal tubular reabsorption, resulting in sustained renal cortical retention and localised radiation exposure. Radiation-induced renal injury is a delayed and progressive process involving glomerular, vascular, and interstitial compartments, with cumulative microvascular damage and fibrosis manifesting months to years after exposure rather than as acute tubular dysfunction [1, 2].

Clinical dosimetry studies have demonstrated that PSMA-targeted ligands differ in renal uptake, with consistently higher kidney absorbed doses reported for [¹⁷⁷Lu]Lu-PSMA I&T compared with [¹⁷⁷Lu]Lu-PSMA-617, likely related to ligand structure and chelator charge [3, 4]. Despite these theoretical concerns, early clinical experience has generally reported low rates of clinically significant nephrotoxicity, particularly in heavily pre-treated metastatic castration-resistant prostate cancer populations with limited life expectancy and relatively short follow-up [5–7].

However, longer-term follow-up studies and detailed renal assessments suggest that subtle but progressive renal impairment may occur with extended treatment exposure or in patients who experience prolonged survival. Recent analyses have reported gradual declines in eGFR over time and, in selected cases, biopsy-proven radiation-associated nephropathy, including thrombotic microangiopathy-like changes following extensive [¹⁷⁷Lu]Lu-PSMA therapy [8, 9]. These observations raise important questions regarding cumulative renal effects that may be underestimated in studies relying on short-term safety analyses.

As PSMA-targeted radioligand therapy is increasingly introduced earlier in the disease course, including prior to chemotherapy and in metastatic hormone-sensitive prostate cancer [10, 11], improved characterisation of time-dependent renal effects and patient-specific susceptibility has become increasingly important.

## Materials and methods

### Study population

This retrospective cohort study included patients treated with [¹⁷⁷Lu]Lu-PSMA I&T at Fiona Stanley Hospital between November 2018 and July 2024. Patients were identified from the institutional Nuclear Medicine database.

Inclusion criteria were: (i) receipt of at least two cycles of [¹⁷⁷Lu]Lu-PSMA I&T during the study period to ensure a minimum level of treatment exposure, (ii) availability of baseline renal function data prior to the first treatment cycle, and (iii) at least one post-baseline eGFR measurement following cycle 1.

Exclusion criteria were the absence of post-treatment renal function data or incomplete treatment records precluding calculation of cumulative administered activity.

All patients were men with metastatic castration-resistant prostate cancer receiving continuous androgen deprivation therapy and had progressed following all available standard systemic therapies, including at least one androgen receptor pathway inhibitor, taxane chemotherapy, and PARP-inhibitor when indicated and accessible. Treatment with [¹⁷⁷Lu]Lu-PSMA I&T was delivered under an institutional compassionate access program following multidisciplinary review.

Imaging-based selection followed institutional criteria consistent with modified TheraP PSMA/FDG PET selection [12]. Eligibility required high PSMA expression on [⁶⁸Ga]Ga-PSMA or [^18^F]-labelled PSMA PET/CT and absence of discordant FDG-avid, PSMA-negative disease on [¹⁸F]FDG PET/CT. PET/CT imaging was performed as part of routine pre-treatment assessment and was centrally reviewed within the Department of Nuclear Medicine prior to treatment initiation.

### Measures

Renal function was assessed using estimated glomerular filtration rate (eGFR), calculated from serum creatinine measurements obtained as part of routine clinical care. eGFR values were calculated using the Chronic Kidney Disease Epidemiology Collaboration (CKD-EPI) creatinine equation (race-free version), consistent with institutional laboratory reporting during the study period.

Baseline renal function was defined as the most recent eGFR value prior to administration of the first cycle of [¹⁷⁷Lu]Lu-PSMA I&T. Follow-up eGFR values were obtained at clinically scheduled intervals corresponding approximately to 3, 6, 12, 24, 36, and 48 months after cycle 1, reflecting routine clinical monitoring rather than protocol-mandated visits.

Baseline renal vulnerability was characterised using a composite Renal Risk Index (RRI), constructed a priori based on established clinical risk factors for chronic kidney disease. The RRI incorporated baseline chronic kidney disease, hypertension, and diabetes mellitus. Chronic kidney disease was defined as a pre-treatment eGFR < 60 mL/min/1.73 m² and assigned a weight of two points. Hypertension and diabetes mellitus were ascertained from electronic medical records based on documented diagnoses and/or active treatment with antihypertensive or glucose-lowering medications at baseline; each was assigned one point. The total RRI score ranged from 0 to 4, with higher scores indicating greater baseline renal vulnerability.

Treatment exposure was quantified using administered radioligand activity per treatment cycle. Activity values were standardised to giga-becquerels (GBq) and summed across cycles to derive cumulative administered activity for each patient. In the absence of patient-specific dosimetry, cumulative administered activity was used to estimate renal absorbed dose using a fixed conversion factor of 0.73 Gy per GBq, derived from published population-level dosimetry data [3]. Estimated renal dose was incorporated into analyses as a continuous variable and scaled per 10 Gy for interpretability.

The primary outcome was longitudinal change in eGFR over time following [¹⁷⁷Lu]Lu-PSMA I&T. A secondary outcome was clinically significant renal function decline, defined as a ≥ 25% reduction in eGFR from baseline.

### Statistical analysis

Statistical analyses were performed using R (version 4.5.2; R Foundation for Statistical Computing). Descriptive statistics were used to summarise patient demographics, treatment characteristics, and baseline renal risk factors. Continuous variables are reported as median with interquartile range (IQR) or mean with standard deviation, as appropriate, and categorical variables as counts and percentages.

Longitudinal changes in renal function were analysed using linear mixed-effects models to account for repeated eGFR measurements within individuals and variable follow-up intervals. Time since the first cycle of [¹⁷⁷Lu]Lu-PSMA I&T (months) was modelled as a fixed effect. Patient-specific random intercepts and random slopes were included a priori to capture heterogeneity in baseline renal function and rates of change over time. Fixed effects were selected based on clinical relevance rather than data-driven selection.

Cumulative administered activity was incorporated as an estimate of renal radiation exposure and expressed as estimated renal absorbed dose, scaled per 10 Gy for interpretability. Renal dose estimates were derived using a population-based activity-to-dose conversion factor, as patient-specific renal dosimetry was not available. Baseline renal vulnerability was modelled using the Renal Risk Index (RRI) as a continuous covariate to reflect graded renal risk burden.

Several prespecified sensitivity analyses were performed to assess the robustness of the primary longitudinal model. First, analyses were repeated after restricting observations to the first 12 months of follow-up to evaluate the influence of sparse later follow-up. Second, potential non-linearity of the time effect was explored by adding a quadratic time term. Third, a piecewise linear mixed-effects model was used in a combined dataset including reconstructed pre-treatment eGFR measurements, allowing separate estimation of pre-treatment and post-treatment slopes relative to therapy initiation.

Mixed-effects models were fitted using maximum likelihood estimation. Missing data were not imputed; mixed-effects modelling inherently accommodates unbalanced longitudinal data under a missing-at-random assumption. Model assumptions were assessed by inspection of residual distributions and random-effects variance components. Model fit was evaluated using Akaike’s Information Criterion (AIC) and Bayesian Information Criterion (BIC). Fixed-effect estimates are reported with 95% confidence intervals (CI) and p-values derived using Satterthwaite’s approximation.

Model-based population-level predictions were generated for visualisation, with individual patient trajectories overlaid to illustrate within- and between-patient variability.

Secondary analyses evaluated the occurrence of clinically significant renal function decline, defined as a ≥ 25% reduction in eGFR from baseline. Time-to-event analyses were conducted using Cox proportional hazards models, with effect estimates reported as hazard ratios and 95% confidence intervals. Kaplan–Meier curves were generated to visualise time to ≥ 25% eGFR decline. Because of the limited number of renal events and substantial competing mortality, these analyses were considered exploratory and interpreted descriptively.

Exploratory analyses also examined the relationship between renal decline and overall survival. To account for immortal time bias, renal decline was modelled as a time-dependent covariate in Cox regression models.

No formal adjustment for multiple comparisons was applied, as analyses were hypothesis-driven and focused on a small number of prespecified covariates. All statistical tests were two-sided, and statistical significance was defined as *p* < 0.05.

## Results

### Study cohort, treatment exposure, and follow-up

**Fig. 1:**
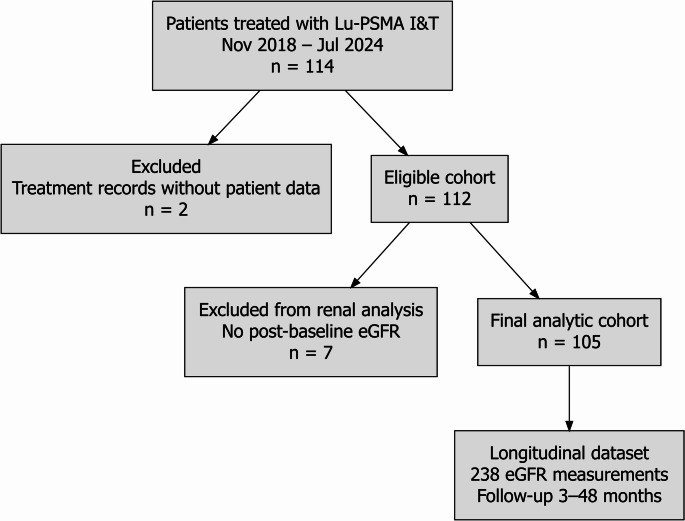
Patient selection and cohort derivationFlow diagram illustrating patient inclusion for the longitudinal renal analysis. A total of 114 treatment records were identified during the study period (November 2018–July 2024). Two records lacked patient identifiers and were excluded. Of the remaining 112 patients, seven were excluded due to absence of post-baseline estimated glomerular filtration rate (eGFR) measurements. The final analytic cohort therefore comprised 105 patients contributing 238 post-treatment eGFR measurements over 3–48 months of follow-up

A total of 112 patients received at least one cycle of [¹⁷⁷Lu]Lu-PSMA I&T during the study period. Of these, 105 met inclusion criteria for longitudinal renal analysis (≥ 2 treatment cycles and ≥ 1 post-baseline eGFR measurement) Fig. 1.

Overall survival data were available for 102 of the 105 included patients. During follow-up, 96 deaths occurred. Median overall survival was 11.8 months (95% CI, 10.1–13.2 months).

Patients received a median of four treatment cycles. The median administered activity per cycle was 8.02 GBq (IQR 7.56–8.40; range 3.68–9.56). The median cumulative administered activity was 31.07 GBq (IQR 17.08–42.83; range 11.13–102.19), corresponding to a median estimated cumulative renal dose of 22.68 Gy (IQR 12.47–31.27), derived using a population-based conversion factor (Table 1).

**Table 1:** Baseline patient characteristics and treatment exposure stratified by Renal Risk Index

Characteristic	Overall (*N* = 105)	RRI ≥ 2 (*N* = 26)	RRI 0–1 (*N* = 79)
Age (years)	74 (66–80)	77 (72–83)	73 (66–80)
Baseline eGFR (mL/min/1.73 m²)	85 (71–90)	53 (49–81)	88 (79–90)
Hypertension	56 (54%)	20 (77%)	36 (46%)
Diabetes mellitus	19 (18%)	16 (62%)	3 (4%)
Baseline CKD (eGFR < 60 mL/min/1.73 m²)	16 (15%)	16 (62%)	0 (0%)
Number of cycles	4 (3–6)	4 (2–5)	4 (3–6)
Cumulative activity (GBq)	31 (22–44)	27 (17–39)	32 (23–44)
Estimated renal dose (Gy)	23 (16–32)	19 (12–28)	24 (17–32)

Values are median (IQR) or n (%)

Renal Risk Index incorporates baseline chronic kidney disease (2 points), hypertension (1 point), and diabetes mellitus (1 point)

Baseline renal function demonstrated moderate variability, with a median baseline eGFR of 85 mL/min/1.73 m² (IQR 71–90; range 39–90) (Table 1).

A total of 238 post-treatment eGFR measurements were available for mixed-effects modelling (median 2 measurements per patient, range 1–6), spanning 3 to 48 months following treatment initiation. Renal observations were concentrated in the early follow-up period. The median maximum follow-up per patient was 6 months (IQR 3–12 months). Attrition over time reflected disease progression and competing mortality. Of the 105 included patients, 41 (39%) remained under observation at 12 months, and 12 (11%) at 24 months Fig. 1.

Baseline renal vulnerability, quantified using the Renal Risk Index (RRI), demonstrated a broad distribution across the cohort: 40 patients had RRI 0, 39 had RRI 1, and 26 had RRI ≥ 2 (Table 1).

Compared with patients in the low-risk group (RRI 0–1), those with RRI ≥ 2 had markedly higher prevalence of diabetes (62% vs. 4%), hypertension (77% vs. 46%), and baseline CKD (62% vs. 0%), consistent with the composite nature of the index (Table 1).

### Post-treatment longitudinal renal function

**Fig. 2:**
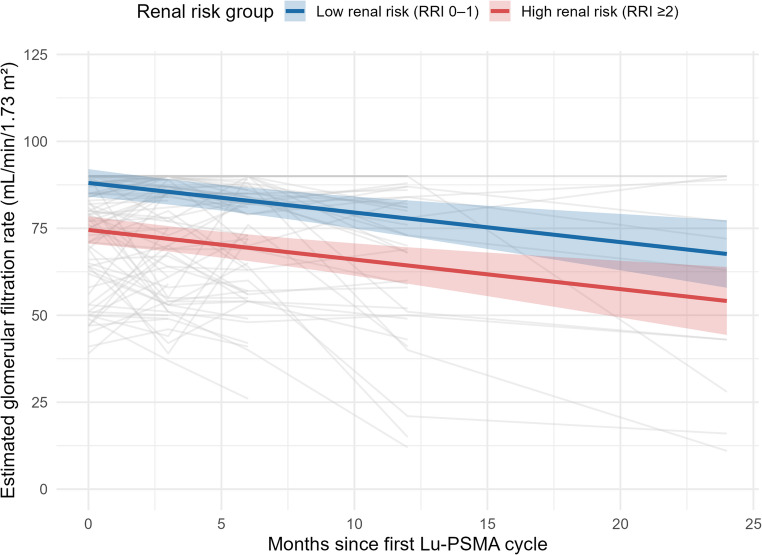
Longitudinal trajectories of estimated glomerular filtration rate (eGFR) following initiation of [¹⁷⁷Lu]Lu-PSMA I&T. Light grey lines represent individual patient trajectories. Coloured curves show population-level predictions derived from the linear mixed-effects model, stratified by Renal Risk Index (RRI) group, with shaded ribbons indicating 95% confidence intervals. Time is expressed as months since the first treatment cycle. The model included random intercepts and slopes for time to account for between-patient variability in baseline renal function and rates of decline. A gradual decline in eGFR over time is observed across both renal risk strata, with lower absolute eGFR levels in patients with higher baseline renal vulnerability but broadly parallel longitudinal trajectories

The primary analysis evaluated longitudinal change in eGFR after treatment initiation using a linear mixed-effects model incorporating random intercepts and slopes (Table 2; Fig. 2). During follow up eGFR declined over time (β = −0.85 mL/min/1.73 m² per month; 95% CI, − 1.29 to − 0.41; *p* < 0.001), corresponding to an annualised decline of approximately 10.2 mL/min/1.73 m² per year.

**Table 2:** Linear mixed-effects model of longitudinal eGFR

Predictor	Estimate	Std. Error	95% CI	p-value
Intercept	88.33	3.46	81.48 to 95.18	<0.001
Time since cycle 1 (months)	−0.85	0.22	−1.30 to −0.40	<0.001
Estimated renal dose (per 10 Gy)	−0.10	1.07	−2.20 to 2.01	0.93
Renal Risk Index (per unit increase)	−6.76	1.24	−9.20 to −4.32	<0.001

Outcome: eGFR (mL/min/1.73 m²)

Random effects: patient-specific intercept and slope for time

Model fit: Observations = 238, Patients = 105

Substantial inter-individual variability in renal trajectories was observed, reflected by significant random slope variance. Baseline renal vulnerability, quantified using the Renal Risk Index (RRI), was independently associated with lower absolute eGFR values (− 6.76 mL/min/1.73 m² per unit increase in RRI; 95% CI, − 9.20 to − 4.32; *p* < 0.001), indicating reduced baseline renal reserve in higher-risk patients. However, RRI was not associated with an accelerated rate of decline over time.

**Fig. 3:**
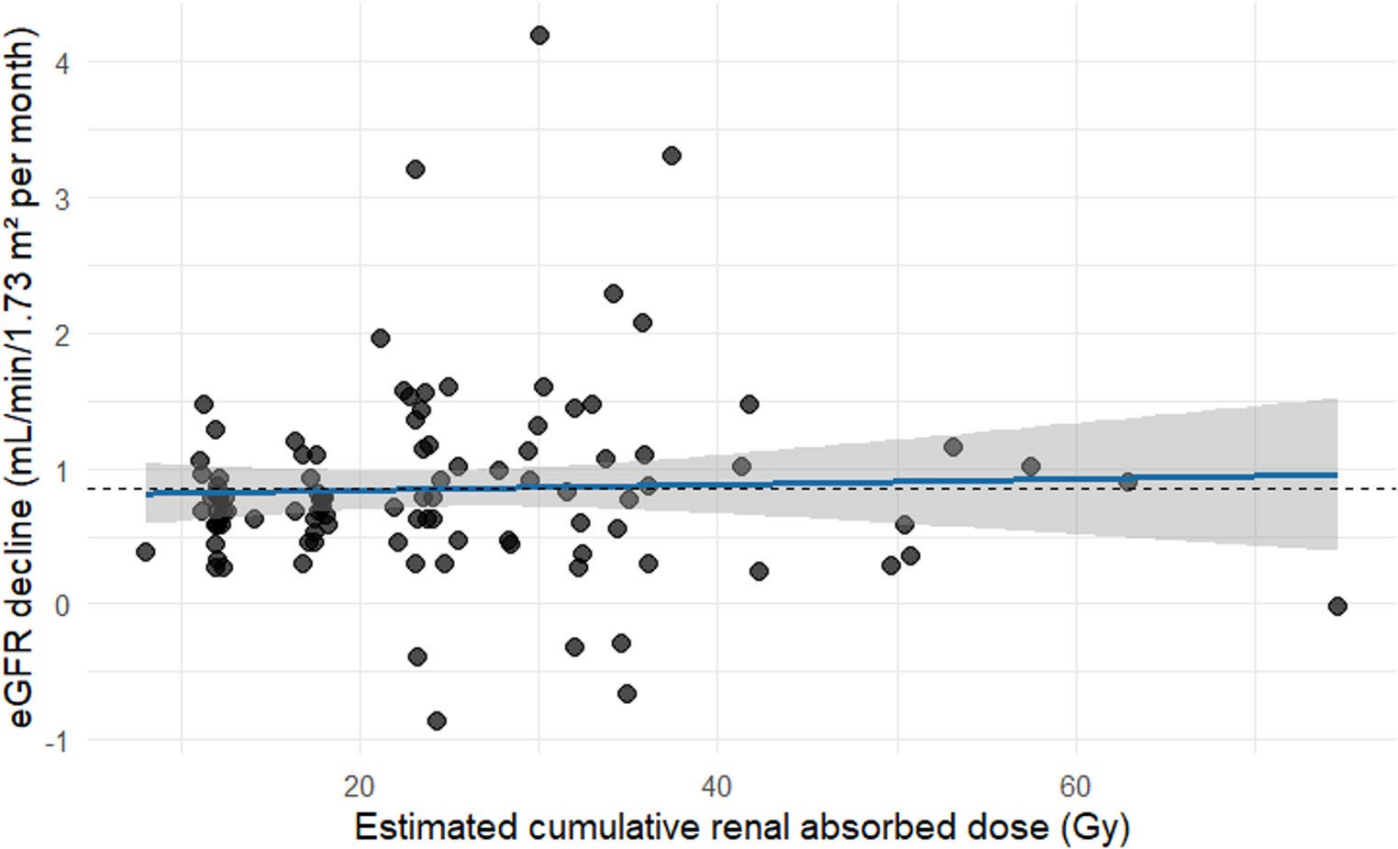
Association between estimated renal absorbed dose and rate of renal function decline. Scatter plot illustrating the relationship between estimated cumulative renal absorbed dose and the patient-specific rate of change in estimated glomerular filtration rate (eGFR). Each point represents an individual patient (N = 105). The y-axis shows the rate of eGFR decline (mL/min/1.73 m² per month), derived from patient-specific slopes estimated from the linear mixed-effects model. The solid line represents the fitted linear regression with 95% confidence interval shading. The dashed horizontal line indicates the average population decline (− 0.85 mL/min/1.73 m² per month) estimated from the primary mixed-effects model. No clear association between estimated renal absorbed dose and the rate of eGFR decline was observed within the administered activity range

Estimated cumulative renal radiation dose (scaled per 10 Gy) was not independently associated with longitudinal eGFR (− 0.10 mL/min/1.73 m² per 10 Gy; 95% CI, − 2.20 to 2.00; *p* = 0.93) within the administered activity range (Fig. 3).

Model fit statistics for the primary model were AIC 1905.2 and BIC 1933.0.

### Pre-treatment renal function trajectory

To contextualise post-treatment changes, reconstructed pre-treatment eGFR data were analysed. Pre-treatment data were available for 92 patients, contributing 1,056 measurements (median 12 measurements per patient; IQR 5–17), spanning a median of 12.0 months prior to treatment initiation (IQR 7.2–12.5 months).

In a piecewise mixed-effects model incorporating both pre- and post-treatment observations (1,245 measurements from 102 patients), eGFR was stable prior to therapy (β = +0.06 mL/min/1.73 m² per month, *p* = 0.30), but declined significantly during follow up after treatment initiation (β = −0.60 mL/min/1.73 m² per month, *p* < 0.001).

### Restricted 12-month and non-linearity analyses

To minimise potential distortion from sparse late follow-up, analyses were repeated restricting observation to the first 12 months post-treatment (*n* = 220 observations). The decline in eGFR remained statistically significant within this period (β = −0.83 mL/min/1.73 m² per month; *p* = 0.002), indicating that the majority of observed decline occurred during the first year.

Assessment of non-linearity using a quadratic time term did not demonstrate significant deviation from linearity (*p* = 0.18), suggesting that the decline in renal function over time was adequately described by a linear trend. A piecewise model separating the first 12 months from later follow-up demonstrated a steeper early decline, with attenuation thereafter; however, the later slope did not reach statistical significance, and estimates beyond 24 months were based on limited numbers at risk.

### Clinically significant renal decline

A ≥ 25% decline in eGFR from baseline occurred in 16 of 105 patients (15%) during follow-up, with most events occurring within the first 12 months.

**Fig. 4:**
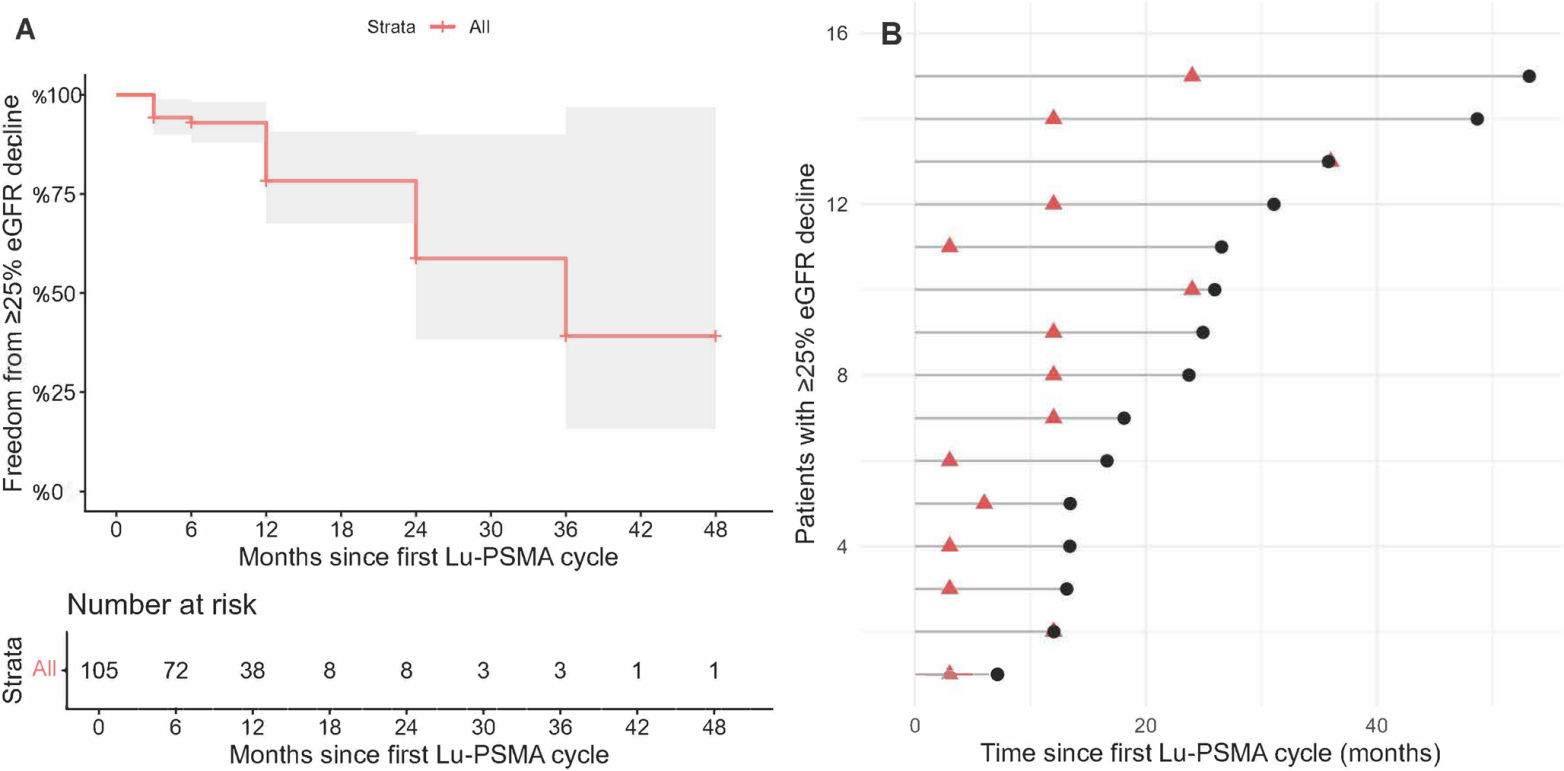
Clinically significant renal decline and its temporal relationship with overall survival. (**A**) Kaplan–Meier estimate of freedom from ≥25% decline in estimated glomerular filtration rate (eGFR) following initiation of [¹⁷⁷Lu]Lu-PSMA I&T (N = 105). Shaded areas represent 95% confidence intervals, with numbers at risk shown below the x-axis. Cumulative incidence estimates beyond 24 months are based on very small numbers at risk (n = 8 at 24 months, declining to n = 1 at 48 months) and should be interpreted with caution. (**B**) Patient-level timelines for individuals who experienced ≥25% eGFR decline and had available survival data (n = 15). Horizontal lines represent overall survival duration from treatment initiation. Red triangles indicate the time of renal decline, and black circles indicate death

Kaplan–Meier analysis estimated a cumulative incidence of ≥ 25% eGFR decline of 22% at 12 months (38 patients at risk) and 41% at 24 months (8 patients at risk) Fig. 4A). Median time to ≥ 25% decline was not reached. Estimates beyond 24 months should be interpreted cautiously due to decreasing numbers at risk and substantial competing mortality.

In exploratory time-to-event analyses, ≥ 25% eGFR decline was not significantly associated with estimated renal dose (HR 0.89 per 10 Gy; 95% CI 0.59–1.34; *p* = 0.57), baseline CKD (HR 0.68; 95% CI 0.14–3.23; *p* = 0.62), or age (HR 0.99 per year; 95% CI 0.93–1.05; *p* = 0.70).

### Renal decline and overall survival

In a conventional Cox model, patients experiencing ≥ 25% renal decline appeared to have longer survival, reflecting immortal time bias inherent to analyses treating decline as a baseline exposure (Fig. 4B). When renal decline was modelled as a time-dependent covariate, its occurrence was strongly associated with mortality (HR 34.5, 95% CI 10.8–110.2, *p* < 0.001). This likely reflects renal deterioration occurring late in the disease trajectory rather than representing treatment-related toxicity.

## Discussion

In this retrospective cohort of patients with advanced metastatic castration-resistant prostate cancer treated with [¹⁷⁷Lu]Lu-PSMA I&T, longitudinal modelling demonstrated a measurable decline in renal function over time within the treated population. The primary mixed-effects model estimated a decline of approximately 0.85 mL/min/1.73 m² per month, corresponding to an annualised reduction of roughly 10 mL/min/1.73 m². Importantly, reconstructed pre-treatment trajectories demonstrated stable renal function prior to therapy, whereas a piecewise model identified a significant post-treatment decline (− 0.60 mL/min/1.73 m² per month) following treatment initiation. While renal function declined during the treatment period in this cohort, the observational design precludes causal attribution of this decline to radioligand therapy itself.

The magnitude of decline observed here exceeds that expected from physiological ageing alone. Longitudinal population studies have consistently reported age-related reductions in glomerular filtration rate of approximately 0.8–1.0 mL/min/1.73 m² per year, including estimates of − 0.75 mL/min/1.73 m² per year in the Baltimore Longitudinal Study of Aging and approximately − 0.9–1.0 mL/min/1.73 m² per year in healthy kidney donors [13–15]. Even among patients with established chronic kidney disease, typical progression rates in large observational cohorts are often reported in the range of 2–4 mL/min/1.73 m² per year [16, 17]. Within this context, the decline observed in the present study appears substantially greater than that attributable to ageing alone. However, given the absence of a comparator group and the advanced disease status of the study population, it remains possible that factors such as systemic illness, cancer progression, or treatment-related comorbidity contribute to the observed renal trajectory.

Baseline renal vulnerability, captured using the Renal Risk Index (RRI), was strongly associated with lower absolute eGFR levels throughout follow-up but did not significantly modify the slope of decline. Longitudinal trajectories were therefore broadly parallel across risk strata. The RRI was included as a composite measure of baseline renal vulnerability, incorporating established risk factors for chronic kidney disease progression, and was intended primarily to explore whether patients with reduced renal reserve experience accelerated decline under treatment. In the present cohort, the absence of slope modification suggests that baseline risk influences starting renal function rather than the rate of subsequent decline. Clinically, this implies that patients with impaired baseline renal function may reach clinically relevant renal thresholds earlier despite similar rates of longitudinal decline.

Estimated cumulative renal radiation dose was not independently associated with longitudinal eGFR decline within the administered activity range studied. This finding should be interpreted cautiously. Renal dose was estimated using a population-based conversion factor rather than patient-specific dosimetry, and the administered activity range was relatively narrow. Under these conditions, the study may have limited ability to detect modest dose–response relationships.

Most previous studies evaluating renal safety of PSMA-targeted radioligand therapy have focused primarily on categorical toxicity grading, generally reporting low rates of grade ≥ 3 nephrotoxicity [7, 12, 18–20]. While reassuring, toxicity grading systems are relatively insensitive to gradual changes in renal function. By modelling eGFR as a continuous longitudinal outcome, the present study demonstrates a measurable decline in renal function over time that may not manifest as overt high-grade toxicity within the relatively limited survival horizons typical of late-line metastatic castration-resistant prostate cancer populations.

Several additional analyses were undertaken to better characterise the temporal pattern of renal change. Restricting the analysis to the first 12 months of follow-up yielded similar slope estimates, indicating that the observed decline is not driven by sparse late follow-up data. Furthermore, testing for non-linear time effects using a quadratic model did not demonstrate statistically significant deviation from linear decline, suggesting that renal function follows an approximately linear trajectory over the observed period. Importantly, however, the effective duration of renal observation in this cohort was limited. The median maximum follow-up per patient was 6 months (IQR 3–12 months), and although follow-up extended to 48 months in a small number of patients, attrition due to competing mortality resulted in rapidly decreasing numbers at risk at later time points.

Clinically significant renal decline, defined as a ≥ 25% reduction in eGFR, occurred in 15% of patients during follow-up. Most events occurred within the first year after treatment initiation. However, cumulative incidence estimates beyond 12 months were based on small numbers at risk and should therefore be interpreted cautiously. Exploratory analyses examining the timing of renal decline relative to survival suggested that when substantial renal deterioration occurred, it often preceded death by a relatively short interval, with a median interval of approximately 3.8 months between renal decline and death. This temporal relationship suggests that renal deterioration in this population may frequently represent a marker of late-stage disease trajectory or global clinical decline, rather than an isolated manifestation of treatment-related nephrotoxicity.

Interpretation of associations between renal decline and survival therefore requires particular caution. In conventional Cox models treating renal decline as a baseline exposure, patients experiencing renal decline appeared to have longer survival, reflecting immortal time bias inherent to this approach. When renal decline was instead modelled as a time-dependent covariate, its occurrence was strongly associated with mortality. This association likely reflects the fact that renal deterioration tends to occur late in the disease course, rather than indicating a causal contribution of renal dysfunction to mortality.

Although the present study was conducted in a late-line mCRPC population with limited survival, these findings may carry greater relevance as PSMA-targeted radioligand therapy moves earlier in the disease course, where patients are expected to survive substantially longer. It must be emphasised, however, that any extrapolation of the observed decline to earlier-line or metastatic hormone-sensitive settings is speculative: the effective follow-up in this cohort was short (median 6 months per patient), observations beyond 24 months are based on very small numbers at risk, and whether renal function continues to decline linearly over longer time horizons remains unproven. These projections should therefore be regarded as hypothesis-generating rather than predictive. Nonetheless, even a modest annualised eGFR reduction could accumulate meaningfully over years of survival, particularly in individuals with limited baseline renal reserve, underscoring the importance of structured longitudinal renal monitoring and prospective evaluation of renal outcomes as radioligand therapy expands into earlier disease settings.

This study is limited by its retrospective design and the requirement for at least two treatment cycles with follow-up renal measurements, which may exclude patients with early deterioration. Renal function was assessed using routine serum creatinine without tubular biomarkers, and renal dose was estimated using a population-based conversion rather than individual dosimetry. High competing mortality also restricted long-term observation and reduced precision at later time points. Nonetheless, the consistency of findings across mixed-effects modelling, restricted follow-up analyses, and piecewise modelling, together with the demonstration of stable pre-treatment renal trajectories, supports the robustness of the observed renal function decline in this treated cohort.

## Conclusion

In this retrospective cohort of patients with late-line metastatic castration-resistant prostate cancer treated with [¹⁷⁷Lu]Lu-PSMA I&T, renal function showed a gradual longitudinal decline during the treatment period despite stable pre-treatment trajectories. Baseline renal vulnerability influenced absolute eGFR but not the rate of decline, and no clear dose–response relationship was observed within the administered activity range. Clinically significant renal deterioration was uncommon and often occurred late in the disease course in the context of substantial competing mortality. These findings highlight the importance of longitudinal renal monitoring and suggest that even modest declines in kidney function may become increasingly relevant as PSMA-targeted radioligand therapy is introduced earlier in the disease trajectory.

## Data Availability

The datasets generated during and/or analysed during the current study are available from the corresponding author on reasonable request.
